# Noise-modulated multistable synapses in a Wilson-Cowan-based model of plasticity

**DOI:** 10.3389/fncom.2023.1017075

**Published:** 2023-02-02

**Authors:** Caroline A. Lea-Carnall, Lisabel I. Tanner, Marcelo A. Montemurro

**Affiliations:** ^1^School of Health Sciences, Manchester Academic Health Science Centre, Faculty of Biology, Medicine and Health, University of Manchester, Manchester, United Kingdom; ^2^School of Mathematics and Statistics, Faculty of Science, Technology, Engineering and Mathematics, The Open University, Milton Keynes, United Kingdom

**Keywords:** Wilson-Cowan model, plasticity, multistability, synapses, homeostatic, functional connectivity, neural mass model

## Abstract

Frequency-dependent plasticity refers to changes in synaptic strength in response to different stimulation frequencies. Resonance is a factor known to be of importance in such frequency dependence, however, the role of neural noise in the process remains elusive. Considering the brain is an inherently noisy system, understanding its effects may prove beneficial in shaping therapeutic interventions based on non-invasive brain stimulation protocols. The Wilson-Cowan (WC) model is a well-established model to describe the average dynamics of neural populations and has been shown to exhibit bistability in the presence of noise. However, the important question of how the different stable regimes in the WC model can affect synaptic plasticity when cortical populations interact has not yet been addressed. Therefore, we investigated plasticity dynamics in a WC-based model of interacting neural populations coupled with activity-dependent synapses in which a periodic stimulation was applied in the presence of noise of controlled intensity. The results indicate that for a narrow range of the noise variance, synaptic strength can be optimized. In particular, there is a regime of noise intensity for which synaptic strength presents a triple-stable state. Regulating noise intensity affects the probability that the system chooses one of the stable states, thereby controlling plasticity. These results suggest that noise is a highly influential factor in determining the outcome of plasticity induced by stimulation.

## 1. Introduction

One hypothesis for the basis for communication in the brain is that it occurs *via* the transient coherence of neuronal assemblies (Fries, 2005). Neural architecture must allow for temporally and spatially distinct sub-networks to form and exchange information on a sub-millisecond timescale. Multistability has been proposed as a potential mechanism underlying this process (Kelso, 2012; Tognoli and Kelso, 2014; Alderson et al., 2020), and is thought to play a crucial role in the brain in the context of numerous cognitive processes and pathologies, including perception, binocular rivalry, auditory stream segregation, and epilepsy (Leopold and Logothetis, 1999; Winkler et al., 2012; Jirsa et al., 2017; Courtiol et al., 2020). There is a growing body of work surrounding the multistable brain; however, there has been minimal focus on the interaction between multistable brain states and their effect on plasticity processes within neural networks. Here we address this open question with a focus on frequency-dependent plasticity which involves applying a periodic stimulus at a specified frequency to elicit changes in connectivity in the brain (Ragert et al., 2008; David et al., 2015; Lea-Carnall et al., 2017, 2020).

Plasticity is the mechanism by which the nervous system adapts to external stimuli over multiple spatial and temporal scales. The phenomenon can manifest in functional or structural changes to neural networks as we experience the world and underlies cognitive processes such as learning and memory. In a previous study, we applied a periodic stimulus at a range of frequencies to a neural network model based on Wilson-Cowan (WC) oscillators connected *via* an activity-dependent learning rule. We showed that there was a relationship between the frequency of stimulation and network connectivity and validated the model against human behavioral and neuroimaging data (Lea-Carnall et al., 2017). However, it is unknown how the multistable states in the dynamics of neural populations can affect plasticity in the brain under entrainment by different stimulation frequencies.

Experimental evidence has shown the existence of both multistability and metastability in biological neural networks (see Tognoli and Kelso, 2014 for a review). The distinction between these is that in a metastable system, the system spontaneously fluctuates between stable states, whereas a multistable system requires exogenous input to drive shifting between the states. On a theoretical level, metastability provides an explanation as to how brain regions can coordinate over a vast range of spatial and temporal scales in order to support cortical function (Bressler and Kelso, 2001, 2016; Jirsa and McIntosh, 2007; Deco et al., 2013). The fact that no energy is required to switch between states protects the brain from becoming trapped in a stationary state which could be pathological.

On the other hand, multistability is ubiquitous in biological systems in general (for example, Crabb et al., 1996; Rietkerk et al., 2004), and particularly in the brain where it is observed across multiple spatial scales from single synapses to neural networks (see Braun and Mattia, 2010). Multistability is hypothesized to underlie human perception (Ditzinger and Haken, 1989; Lumer et al., 1998; Haynes et al., 2005; Sterzer et al., 2009) (see also Freyer et al., 2011), decision making (Deco and Rolls, 2006), behavior (Schöner and Kelso, 1988), audition (Kondo et al., 2017), and motor dynamics (Jantzen et al., 2009). The term refers to the existence of attractor states, which in the brain can be thought of as stable patterns of activity which persist for some period of time and to which the system returns to again after perturbation (Kelso, 2012). It is thought that neural assemblies switch their activity between stable states in response to specific network tasks providing a mechanism for self-organization (Kelso, 1995, 2012). In terms of learning and plasticity, it is currently unknown how a multistable dynamical landscape affects plasticity processes. Changes in connectivity may allow the network access to new attractor states or may stabilize or destabilize existing ones. An understanding of the biophysical implications of the interaction between these two critical features of neural networks would have fundamental consequences in allowing us to optimize stimulation paradigms to maximize plasticity outcomes. Theoretical work has shown that the WC model, as well as many mathematical neural models, exhibits multi- and metastability both at rest and in response to stimulation (Golos et al., 2015; Deco et al., 2017; Zhang et al., 2022), which makes it an ideal test model to investigate the effects of multistable plasticity regimes.

Moreover, noise in dynamical systems can have a significant role in determining the stable-state landscape and in allowing transitions (switching) between those states. Therefore, our aim was to characterize synaptic plasticity dynamics in a model of interacting neural populations as a function of stimulation frequency and noise intensity. To that purpose, we implemented a WC model of neural activity that included activity-dependent and homeostatic plasticity mechanisms.

## 2. Methods

### 2.1. Wilson-Cowan units

The mathematical details describing the time-evolution of the activity for the E and I populations are given in Equations (1–3). In the equations, the subindex *i* refers to a reference unit while the subindex *j* runs over the rest of the units in the network, and the parameters without subindices have the same value for all units.


(1) $$\begin{array}{l} \tau_{E}\frac{dE_{i}}{dt}=-E_{i}+S(W_{EE}E_{i}+W_{EI}I_{i}+E_{0}\\ \;+\underset{j\neq i}{\sum }w_{ij}E_{j}+f\left(t\right)+z\xi_{i}\left(t\right)-S_{E,i}) \end{array}$$

(2) $$\begin{array}{l} \tau_{I}\frac{dI_{i}}{dt}=-I_{i}+S\left(W_{IE}E_{i}+W_{II}I_{i}+I_{0}+\underset{j\neq i}{\sum }u_{ij}E_{j}-S_{I,i}\right) \end{array}$$

(3) $$\begin{array}{l} S\left(x\right)=\frac{1}{1+e^{-m\left(x-n\right)}} \end{array}$$

**Figure 1:**
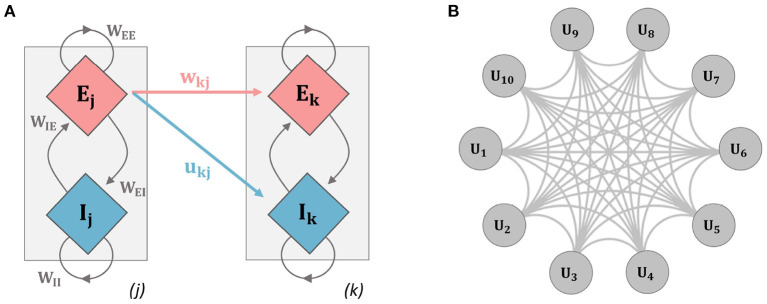
Schematic diagrams for the networks used in analysis. **(A)** The two-unit network used throughout the paper (units *i* and *j* refer to units 1 and 2 here, but indices were kept to relate explicitly to the general model equations): a single unit contains an excitatory (E) and inhibitory (I) population of neurons, inter-population connectivity strengths are described by the parameters *W*_*EE*_, *W*_*IE*_, *W*_*II*_, and *W*_*EI*_. Inter-unit connection strength is governed by connectivity parameters contained in the connectivity matrices *w*_*jk*_ and *u*_*jk*_. Mathematical details for the model dynamics are given in Equations (1–6). **(B)** Ten-unit model: each unit contains an E and I population as in the previous example, the network is fully connected with each gray connection indicating a bi-directional E and I connection.

Where *E*/*I* denotes the activity of the excitatory/inhibitory population for each mass, the strength of intra-unit connectivity is governed by the parameters *W*_*EE*_, *W*_*EI*_, *W*_*IE*_, *W*_*II*_. The model also receives zero-mean and unit-variance white-noise input, ξ_*i*_(*t*) which is independent for each unit, and a deterministic input given by *f* (*t*) which is the sinusoidal input applied at the same frequency and phase to all units. The parameter *z* sets the standard deviation of the noise input. In all simulations, *W*_*EE*_ = 23, *W*_*II*_ = 0, *W*_*IE*_ = 35, *W*_*EI*_ = 15, as these have been shown to generate stable oscillatory behavior when coupled with the background activity levels given below, see Wang et al. (2014). The sigmoidal function S, is given in Equation (3) which is increasing in the interval *x* ∈ (−∞, ∞); *m* controls the steepness of the curve, and *n* the offset from 0. These parameters were fixed as *m* = 1, *n* = 4 so as to generate stable oscillations as in Wang et al. (2014). Background activity levels were controlled by the parameters *E*_0_, *I*_0_ for the E and I populations respectively and fixed as *E*_0_ = 0.5, *I*_0_ = −5, as in Lea-Carnall et al. (2016). The homeostatic scaling parameters *S*_*E*/*I*_, and the weights *w*_*ij*_ controlling excitatory connections are described in the next section. A schematic of the models are given in Figure 1.

The WC unit has an intrinsic resonance frequency, *f*_*r*_, that is controlled *via* the excitatory and inhibitory time constants τ_*E*_ and τ_*I*_ (Wang et al., 2014). The time constants were varied for each condition to tune the units to a specific internal resonance frequency. The specific values used were *f*_*r*_ = 4 Hz: τ_*E*_ = 0.017 s, τ_*I*_ = 0.013 s, *f*_*r*_ = 8 Hz: τ_*E*_ = 0.024 s, τ_*I*_ = 0.014 s, *f*_*r*_ = 12 Hz: τ_*E*_ = 0.011 s, τ_*I*_ = 0.007 s, and *f*_*r*_ = 23 Hz: τ_*E*_ = 0.014 s, τ_*I*_ = 0.006 s, these were derived computationally.

### 2.2. Synaptic dynamics and homeostatic scaling within the model

Inter-unit connectivity structure and strength are contained in the quantities *w*_*ij*_ and *u*_*ij*_. In particular, all connections to the inhibitory populations are set *u*_*ij*_ = 0.1. For the connections to the excitatory population, an initial value is set for *w*_*ij*_ = 0.15 at *t* = 0. Connections to the excitatory populations are plastic and evolve according to an activity-dependent learning rule. Two different model architectures were used. We first implemented a two-unit model, with unit 1 receiving synapses from unit 2. Finally, a ten-unit network was implemented where units were connected *via* all-to-all coupling and the initial connection strengths were set as stated above (see Figure 1).

Equation (4) specifies the learning rule which governs the dynamics of the excitatory connections *w*_*ij*_. In order for *w*_*ij*_ to increase, the product of the rates *E*_*i*_ and *E*_*j*_ must be greater than a threshold *h*, which results in a positive contribution to the rate of change of *w*_*ij*_ and the synapse is enhanced. Conversely, if *E*_*i*_ = *E*_*j*_ = 0 or if the product of the fractional firing rates *E*_*i*_ and *E*_*j*_ is less than the threshold *h*, then *w*_*ij*_ will decay toward zero. The nonlinear threshold is implemented by the Heaviside function Θ(*x*), which is zero for *x* ≤ 0 and 1 otherwise. Equations (5, 6) describe the homeostatic scaling processes developed by Remme and Wadman (2012) which scale the activity of both populations to maintain their activity within a desired range. The value for *S*_*E*/*I*_ is calculated for each population for each unit and is subtracted from the population's activity at each point in time, see Remme and Wadman (2012) for details.


(4) $$\begin{array}{l} \tau_{h}\frac{dw_{ij}}{dt}=-w_{ij}+\gamma E_{i}E_{j}\Theta \left(E_{i}E_{j}-h\right) \end{array}$$

(5) $$\begin{array}{l} \tau_{S_{E}}\frac{dS_{E,i}}{dt}=E_{i}-E_{\infty } \end{array}$$

(6) $$\begin{array}{l} \tau_{S_{I}}\frac{dS_{I,i}}{dt}=I_{i}-I_{\infty } \end{array}$$

Where τ_*h*_ = 2.5 s, γ = 1, *E*_∞_ = 0.2, *I*_∞_ = 0.2, τ_*S*_*E*__ = 1 s, τ_*S*_*I*__ = 2 s, and *h* = 0.04, as in Lea-Carnall et al. (2017). The parameter *h* was chosen as the square of the mean fractional firing rate for the excitatory population in response to white noise. Therefore, in order for the connectivity to increase between two units, at least 50% of the excitatory population of each of the connected units must be firing; τ_*h*_ and γ were chosen so that the slope of the decay (when the two units were not coincidentally firing) was equal to that of the increase, as in Lea-Carnall et al. (2017). The learning rate, decay rate, and homeostatic processes all act on timescales of seconds. The first has been shown to be biologically relevant (Zenke and Gerstner, 2017) requiring the latter parameters to act on the same scale as a computational necessity to avoid runaway plasticity (Zenke et al., 2017).

### 2.3. Model input and integration

Each unit within the network received an independent Gaussian white-noise input, ξ (*t*), then scaled by a multiplicative factor, *z*, which was varied across conditions.

In the case of the rhythmic input *f* (*t*) we generated a sine wave at the desired frequency with a height of 0.5 and applied a jitter to the start time within the first 1,000 ms to cancel out any phase interactions of the input with the model's endogenous oscillations.

The Euler-Murayama method was used for the integration of stochastic differential equations with an integration step of 1 ms (Higham, 2001). For each experiment we ran 100 trials unless specified otherwise. For each trial we allowed the model to run for 5 × 10^5^ time steps in the case of the 2-unit model and 10^6^ time-steps for the 10-unit model. The results given in **Figures 5**, **7** relate to a single trial and so the simulation ran for longer to illustrate the model behavior. In the first instance, the model ran for 10^6^ time-steps and the first 3 × 10^5^ were discarded, while in the second, the model ran for 2 × 10^6^ time-steps and 1 × 10^5^ were discarded. Transmission delays were assumed to be instantaneous, although we note that in models of STDP, delays within the network have been shown to influence plasticity outcomes (Madadi Asl et al., 2018a,b).

### 2.4. Other methods

Initially, each time series was band-pass filtered ± 5 Hz around *f*_*r*_, Hilbert transformed, and then the instantaneous phase was extracted for each point in time *n* over the window of length *N*.

The complex phase locking value (*cPLV*) is defined as follows,


(7) $$\begin{array}{l} cPLV\equiv PLVe^{i\Phi }=\frac{1}{N}\overset{N}{\underset{n=1}{\sum }}e^{i\Delta \phi \left(n\right)}, \end{array}$$

Where Δϕ(*n*) is the instantaneous phase difference ϕ_1_(*n*)−ϕ_2_(*n*) at each discrete time point *n* between the two filtered signals. The modulus of the *cPLV* is the real phase locking value (*PLV*), which is a measure of phase locking (or synchrony) between two time series. If the two signals are phase-locked, then *PLV* will be close to 1 and it is smaller otherwise, Lachaux et al. (1999). The phase Φ of the *cPLV* provides an average phase difference between the unit's individual phases, as in Petkoski et al. (2018).

In **Figures 5E**, **F**, the simulated time-series data for the two units was segmented according to whether $\overset{-}{w}$ was in the “low,” “mid,” or “high,” regime. This was achieved by applying a cut-off to the weight parameter of $\overset{-}{w}<$ 0.01 for “low,” $0.025<\overset{-}{w}<0.0275$ for “mid,” and $\overset{-}{w}>0.06$ for “high” and then and then calculating the phase difference between the time series as well as the phase of *cPLV* pertaining to each state.

The Kuramoto order parameter (Kuramoto, 1984; Daffertshofer et al., 2018; Petkoski et al., 2018) quantifies the degree of order over a network of phase oscillators, and is defined as,


(8) $$\begin{array}{l} \zeta \left(t\right)\equiv r\left(t\right)e^{\psi \left(t\right)}=\frac{1}{K}\overset{K}{\underset{i=1}{\sum }}e^{\phi_{i}\left(t\right)}, \end{array}$$

Where *K* is the size of the ensemble. The quantity *r*(*t*) is a measure of the spatial global coherence of the ensemble at each time point, while ψ(*t*) is an instantaneous average phase. In **Figure 7** we used the modulus of the Kuramoto order parameter, *r*(*t*), as a measure of instantaneous global coherence.

All simulations were performed in Matlab (The Mathworks Inc., MATLAB ver. R2019b).

## 3. Results

The WC model, a neural mass model, is used to represent each unit (or mass) within our network (Wilson and Cowan, 1972, 1973). Each unit can produce a range of behaviors, including oscillations and evoked responses, depending on the choice of parameters. A “unit” consists of two interconnected populations; one excitatory (*E*) and one inhibitory (*I*). The output of the WC model is a fractional firing rate of the E-population and is interpreted as the proportion of cells within that population that is firing at any point in time. Inter-unit connectivity structure and strength are contained in the quantities *w*_*ij*_, for excitatory connections and *u*_*ij*_, for inhibitory connections which are fixed connecting unit *j* to unit *i*. Inter-unit connections between excitatory populations are plastic and evolve according to an activity-dependent learning rule. In the results that follow, we examine the interaction between the resonance frequency of the model, the frequency of stimulation, and the level of additive noise and assess the effect these have on the multistable states of the plastic excitatory connection strengths.

### 3.1. Effect of stimulation frequency on connection strength

**Figure 2:**
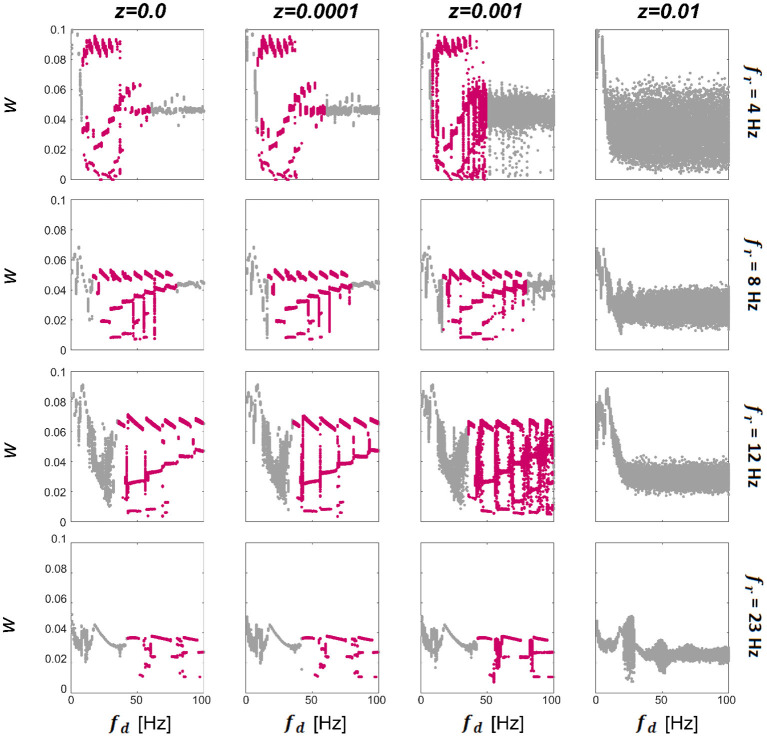
Effect of noise and driving frequency on connection strength. The effect of driving frequency (*f*_*d*_) and noise (*z*) on the synaptic strength *w*. We display the final *w* (each dot represents a single trial) between 2 units in response to a driving frequency presented between 1 and 100 Hz at a range of noise levels for networks tuned to have intrinsic resonance values (*f*_*r*_) of 4 Hz **(top panel)**, 8 Hz, **(second panel)**, 12 Hz **(third panel)** and 23 Hz **(bottom panel)**. It can be seen that for additive noise values shown here of less than z = 0.01 (left 3 columns), there is a region of the plot that exhibits multistable behavior (highlighted in pink), which begins at driving frequencies close to the resonance of the network and collapses at approximately ten times this value. For the **lower two panels**, the multistability initially collapses to a bi-stability and finally to a single attraction point. For all networks, when noise levels are raised sufficiently high, the multistability is abolished altogether. Within the multistable regions, there is further structure evidenced by the staircase-style pattern which repeats at approximately every 0.5 of the resonance.

We first consider a model consisting of two WC units, with *w*_12_ and *u*_12_ being the only non-zero synapses. Since there is only one excitatory weight *w*_12_, from this point we omit the subscript and refer to it as *w*. To characterize the synaptic dynamics of the model, we first computed the final strength of the excitatory synapse as a function of the driving (stimulation) frequency (*f*_*d*_) for different resonant frequencies of the units (*f*_*r*_) and levels of noise intensity (*z*), see Methods for details of how these parameters were computed. The final connection strength (*w*) between the units per condition are presented in Figure 2. We observe that for zero and low levels of noise (left three columns in Figure 2) there exist multistable states (highlighted in pink; each dot represents a single trial) that begin approximately when the driving frequency reaches double the resonance frequency of the system. Then when the value of the driving frequency is approximately 10 times the resonance frequency of the system, there is a transition either to a bi-stable state or a single state that becomes broader for low *f*_*r*_ frequencies. There is a cut-off point for *z* beyond which there are no multistable states for any combination of *f*_*r*_ and driving frequency (*f*_*d*_). Within the multistability, there is further structure evidenced by the appearance of a staircase-like pattern, with sub-structures that repeat at approximately every first sub-harmonic of the resonance (*f*_*r*_). The multistability appears for all values of *f*_*r*_ shown, indicating that this behavior could be present in different regions of the human brain known to exhibit a preferred resonance frequency (Galambos et al., 1981; Snyder, 1992; Herrmann, 2001). We also find that the range of *f*_*d*_ in which multistability occurs extends further for systems with higher resonance frequencies (*f*_*r*_). The resonance frequencies chosen here are 4 (delta), 8 (theta), 12 (alpha), and 23 (beta) Hz; broadly chosen to represent the natural dominant frequencies found in the brain. To see the temporal evolution of *w* over a range of *z* demonstrating multistable plasticity, please see Supplementary Information.

### 3.2. Effect of additive noise on connection strength

**Figure 3:**
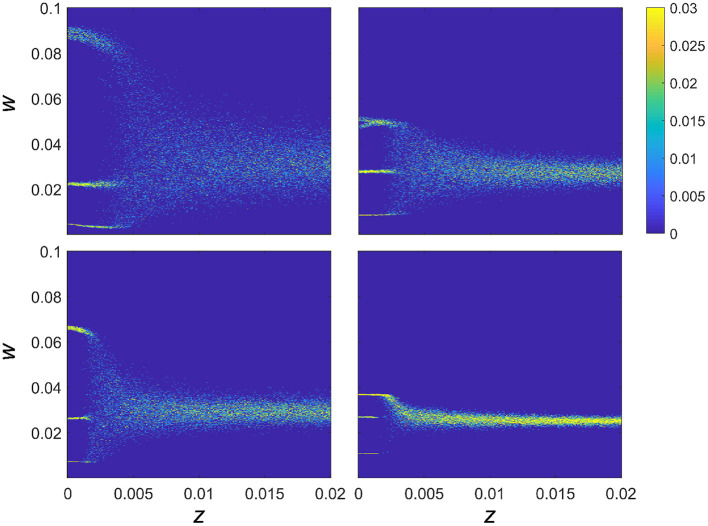
Effect of noise on connection strength with fixed resonance and driving frequency. The effect of noise on multistable plasticity in the WC model. *P*(*w*|*z*) is given as a function of increasing noise for 4 separate networks with fixed resonances and a fixed driving frequency chosen to highlight the multistable behavior of the connection strengths. Resonance and driving frequency combinations are: **top left** *f*_*r*_ = 4 Hz, *f*_*d*_ = 20 Hz; **top right** *f*_*r*_ = 8 Hz, *f*_*d*_ = 32 Hz; **lower left** *f*_*r*_ = 12 Hz, *f*_*d*_ = 48 Hz; **lower right** *f*_*r*_ = 23 Hz, *f*_*d*_ = 86 Hz.

In order to explore the effect of synaptic noise on the dynamics of the synaptic strength, we analyzed the behavior of the connectivity strength for four fixed combinations of intrinsic resonance (*f*_*r*_) and driving frequency (*f*_*d*_), for *z* varied between 0 and 0.02, as shown in Figure 3. In particular, for each value of the noise intensity, *z*, and synaptic strength *w*, Figure 3 shows the color-coded probability of the weight for each given value of the noise as *P*(*w*|*z*). It can be seen that for each of the 4 combinations of *f*_*r*_ and *f*_*d*_ (chosen so as to highlight one of the multistable regions) that the multistable states exist for low levels of additive noise. In all cases, there is a critical value of the noise intensity after which there is a transition to a single broad region of attraction that tends to narrow down for higher levels of noise. Moreover, within the multistability regions shown in Figure 3, noise also affects *P*(*w*|*z*) and therefore the intrinsic structure of the multistable synaptic states, albeit in a subtle manner, as we detailed below.

**Figure 4:**
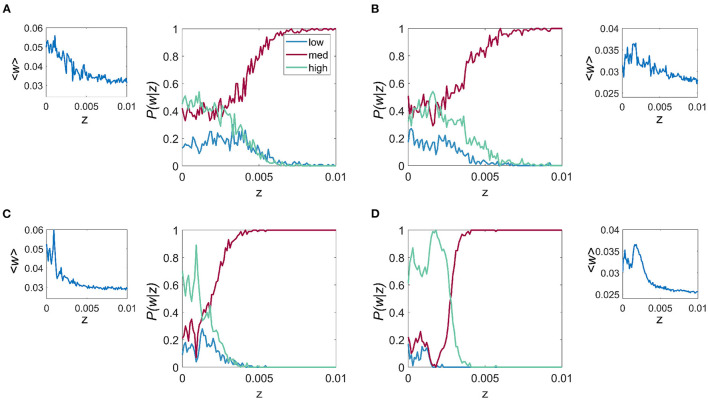
Effect of noise on the probability of connection strength with fixed resonance and driving frequency. *P*(*w*|*z*) is given for each of the 3 connection strength states “low,” “mid,” and “high,” main figure, and the mean connection strength over all trials as a function of the noise, *z* for the *f*_*r*_/*f*_*d*_ combinations of **(A)** *f*_*r*_ = 4 Hz, *f*_*d*_ = 20 Hz, **(B)** *f*_*r*_ = 8 Hz, *f*_*d*_ = 32 Hz, **(C)** *f*_*r*_ = 12 Hz, *f*_*d*_ = 48 Hz, and **(D)** *f*_*r*_ = 23 Hz, *f*_*d*_ = 86 Hz. It is apparent that for all cases, lower levels of noise result in higher mean connection strength and also the highest probability of attaining the high *w*.

There are two key aspects to explore further in relation to the results in Figure 3. The first regards the overall behavior of the trial averaged synaptic strength, 〈*w*〉, which is depicted in Figure 4 (inset), and shows the overall tendency of noise to be deleterious in the case of frequency-dependent plasticity. In particular, for higher values of *z*, there is a decreasing trend in synaptic strength. At lower values of *z*, where the discrete multistable states are present, the behavior of 〈*w*〉 has a more complex structure, which is a consequence of a nontrivial interplay between the noise and the relative stability of the three possible equilibrium states.

To characterize the role of noise on the relative probability of the system choosing a particular synaptic strength state *k*, with *k* = 1, 2, 3 (i.e. “low,” “mid,” and “high” states), we studied the probability *P*(*w*_*k*_|*z*), for a range of values of *z* and for the same four *f*_*r*_ and *f*_*d*_ combinations described previously. We observe that for all cases, especially those cases with higher *f*_*r*_, which include Figures 4C, D, when *z* is less than the critical point where the multistable states collapse (*z*_*c*_), it is more probable that *w* will be that of the “high” state. For *z* > *z*_*c*_, the three states collapse into a single state which is close in value to the mid-state *w*. The results also show that within the multistability region, noise affects the balance of the relative probability of each of the possible states.

### 3.3. Synaptic strength and correlation of activity across units

In what follows, a generic excitatory synaptic weight *w*_*ij*_ will be represented as *w* to simplify notation. The equation modeling the plasticity in our two-unit model is:


(9) $$\begin{array}{l} \tau_{h}\frac{dw}{dt}=-w+\gamma E_{i}E_{j}\Theta \left(E_{i}E_{j}-h\right) \end{array}$$

Let us take a time-average of the above differential equation over a coarse-grained time interval Δ >> τ_*h*_. Thus, we have:


(10) $$\begin{array}{l} \tau_{h}\frac{d\bar{w}}{dt}=-\bar{w}+\gamma \bar{E_{i}E_{j}\Theta \left(E_{i}E_{j}-h\right)} \end{array}$$

Where we used the fact that time averaging and differentiation commute, as shown below. For simplicity, we also assumed that during the time Δ the product of the activities was above the threshold. Let's notice that $\bar{E_{i}E_{j}\Theta \left(E_{i}E_{j}-h\right)}$ is also a function of time, so in principle, we cannot solve the equation without solving the whole model. However, If we also assume that Δ is shorter than the typical time scales of changes in the correlation between the activities, we can replace the average $\bar{E_{i}E_{j}\Theta \left(E_{i}E_{j}-h\right)}$ with a constant *C*_Δ_ indicating the threshold correlation within the time-window Δ. Therefore,


(11) $$\begin{array}{l} \tau_{h}\frac{d\bar{w}}{dt}=-\bar{w}+\gamma C_{\Delta } \end{array}$$

We can now solve the equation within the time-window Δ. The solution is:


(12) $$\begin{array}{l} \bar{w}=w_{0}e^{-\frac{t}{\tau_{h}}}+\gamma C_{\Delta }\left(1-e^{-\frac{t}{\tau_{h}}}\right) \end{array}$$

Where *w*_0_ is the value of the synaptic strength at the start of the interval Δ. Since we assumed that Δ ≫ τ_*h*_ we can study the above solution for times of order Δ that satisfy Δ > *t* >> τ_*h*_. Thus, in that regime the exponentials are very small and can be neglected, leading to the final approximation,


(13) $$\begin{array}{l} \bar{w}=\gamma C_{\Delta } \end{array}$$

Therefore, we conclude that the coarse-grained average of the synaptic strength is proportional to the correlation *C*_Δ_, with proportionality constant equal to γ. This result is consistent with Hebbian-like synaptic dynamics stating explicitly that synaptic strength between two units is directly proportional to the correlation between the activity of such units.

We now show that the time averaging and differentiation commute. Without any loss of generality, we assume that the time average can be written as a convolution with an appropriate kernel. That is,


(14) $$\begin{array}{l} \bar{w}=\int_{-\infty }^{\infty }w\left(s\right)G\left(t-s\right)ds \end{array}$$

Then, we take the derivative with respect to *t* of the expression above and then integrate by parts, obtaining


(15) $$\begin{array}{l} \frac{d\bar{w}}{dt}=\int_{-\infty }^{\infty }w\left(s\right)G^{\prime }\left(t-s\right)ds \end{array}$$

(16) $$\begin{array}{l} ={\left[w\left(s\right)G\left(t-s\right)\right]}_{-\infty }^{\infty }+\int_{-\infty }^{\infty }\frac{dw}{ds}G\left(t-s\right)ds \end{array}$$

(17) $$\begin{array}{l} =\bar{\frac{dw}{dt}}\;, \end{array}$$

Where the first term in Equation (16) is zero due to the kernel finite support and we used the prime as a short-hand notation of the time derivative of the kernel *G*(*t*).

### 3.4. Effect of connection strength correlation, synchrony, and phase-space dynamics

**Figure 5:**
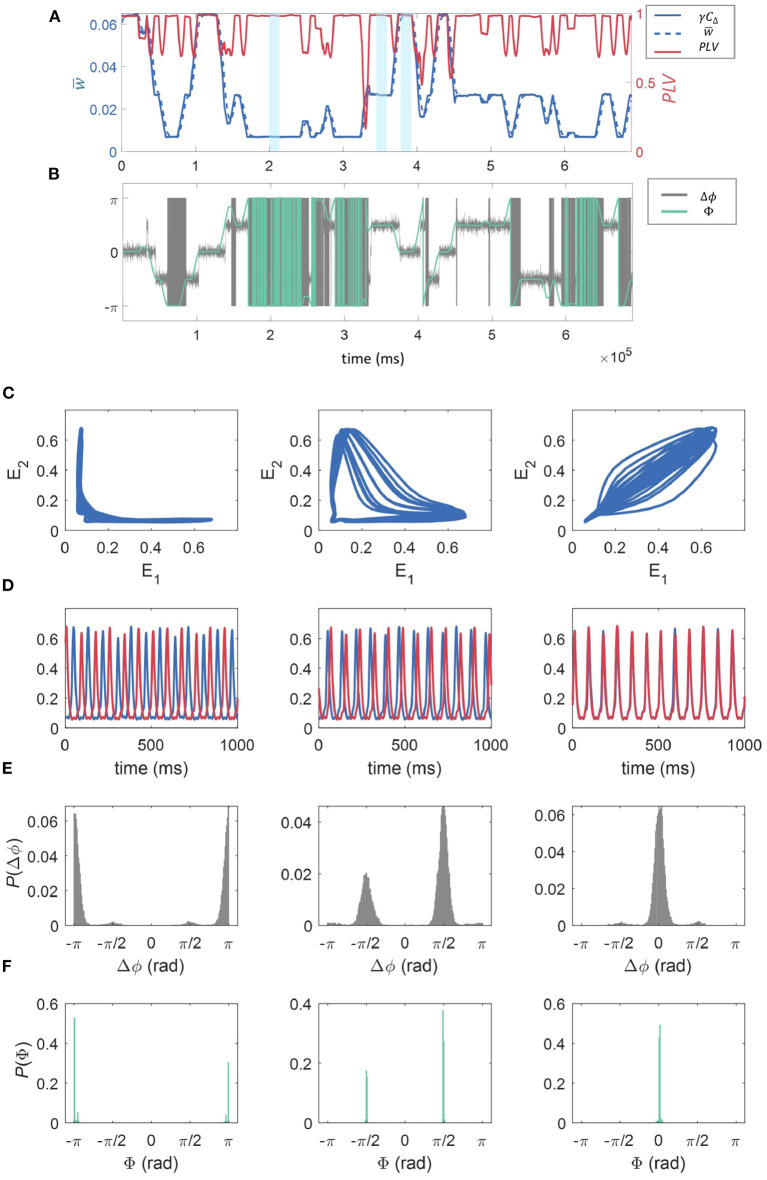
The effect of connection strength on correlation, synchrony, and phase-space dynamics. **(A)** Shows the time evolution of γ*C*_Δ_, where $C_{\Delta }=\bar{E_{1}E_{2}\Theta \left(E_{1}E_{2}-h\right)}$ in blue (solid line, left axis), $\bar{w}$ in blue (dashed line, left axis), and the phase locking value, *PLV*(*t*) between the two excitatory populations in red (right axis). These were all calculated over a sliding window of 10,000 time-steps. The time-averaged synaptic strength $\bar{w}$ can be seen to switch between three states (“low,” “mid,” and “high,” three examples highlighted in yellow) and its temporal evolution is closely matched by the truncated correlation between the time series—see Methods. The *PLV* is generally close to 1, reducing momentarily at the points where the states “switch.” **(B)** The temporal evolution of the phase difference (Δϕ) for the 2 units is given in gray, and the phase of the *cPLV*(Φ) in green. **(C)** *E*_1_ plotted against *E*_2_, and the time series for the fractional firing rates *E*_1_ and *E*_2_ (*E*_1_ is blue, *E*_2_ is red) for the three $\bar{w}$ regimes “low” **(C)**, “mid” **(D)**, and “high” **(E)** relating to 1,000 ms of model activity highlighted in light blue in **(A)**. **(D)** The time series for the fractional firing rates *E*_1_ (blue) and *E*_2_ (red) for the same sections of the simulation as in **(C)**. We see low synchronization between the units for low $\bar{w}$ moving to high synchronization for the high $\bar{w}$ regime. Finally, the probability density histograms for the phase difference between the two units (Δϕ), and the phase of the *cPLV*(Φ), for each of the weight states “low” (left), “mid” (middle), and “high” (right), calculated across the entire simulation are given in **(E, F)**.

As shown in Section 3.3, within the synaptic dynamics incorporated in our model a direct proportionality is expected on average between the correlation of activity between the sub-units' excitatory populations and synaptic strength. This is supported by the results shown in Figure 5A. Here we have fixed *f*_*r*_ = 12 Hz, *f*_*d*_ = 48 Hz, and *z* = 0.0015 to investigate a specific example of the effect of connection strength on synchrony between the units. This result directly links the multistability in synaptic strengths to overall multistability in the correlation dynamics between the WC units. Moreover, the correlation traces shown in Figure 5A clearly show the presence of the three possible states of the dynamics in temporal succession, which in turn correspond to the synaptic states as related by the discussion in Section 3.3. We find that correlation between the units is temporally associated with the synaptic state in that high correlation coincides with “high” *w*, low correlation coincides with low *w*, with “mid” *w* being somewhere in between (see Figure 5A). Also in panel (a), the *PLV* between the two units is shown as a function of time for a realization of the dynamics. The *PLV* stabilizes at a value of 1 at each of the stable states, with a value less than 1 only at the transition between states. This indicates that for all stable states, units are phase-locked. Below, we show that the modulation in signal correlation across states is due to state-dependent phase shifts.

To better understand these relationships, in Figure 5B, we plotted the phase difference between the 2 units, Δϕ, in gray as well as Φ, the phase of the *cPLV*, in green.

The relationship between the three stable states with both the correlation and synchrony suggests a nontrivial relationship in the phase-space dynamics of the excitatory populations. In Figure 5B we show the relation between the main dynamics variables characterizing the state of the excitatory populations, *E*_1_ and *E*_2_, in the “low” (left), “mid” (middle), and “high” (right), synaptic strength states respectively marked in blue in Figure 5A. Consistently with the previous results, Figure 5 when the synaptic strength is “low” we observe weak correlations between the values of *E*_1_ and *E*_2_. In particular, for most of the trajectory either variable remains almost fixed, while the other changes over a range of values. In the “mid” synaptic state, the trajectory in the phase space of both *E*_1_ and *E*_2_ detaches further from the axes leading to slightly higher correlations. Finally, on the right, we observe behavior consistent with the high correlation and phase locking values associated with the higher synaptic state: both *E*_1_ and *E*_2_ follow a cycle of high eccentricity aligned close to the diagonal in the *E*_1_, *E*_2_ plane. In Figure 5D we show examples of the temporal traces for both units for each of the corresponding states (related to the portion of the time series highlighted in blue in Figure 5A). The phase relationships are consistent with the phase-space trajectories discussed above, with increasing similarity in the phase from left to right.

In Figure 5E, we give the probability density histograms for the phase difference between the two units (Δϕ), and in Figure 5F the phase of the *cPLV* (Φ), for each of the weight states “low” (left), “mid” (middle), and “high” (right), calculated across the entire simulation. We observe that when the synaptic strength is in the “low” state, the phase differences are ±π, indicating that the signals are anti-phase, as observed in Figure 5D. In the “mid” state, the phase differences are distributed between ±π/2, and for the “high” state, the phase differences are centered around zero, indicating synchrony.

### 3.5. Multi-unit network model

**Figure 6:**
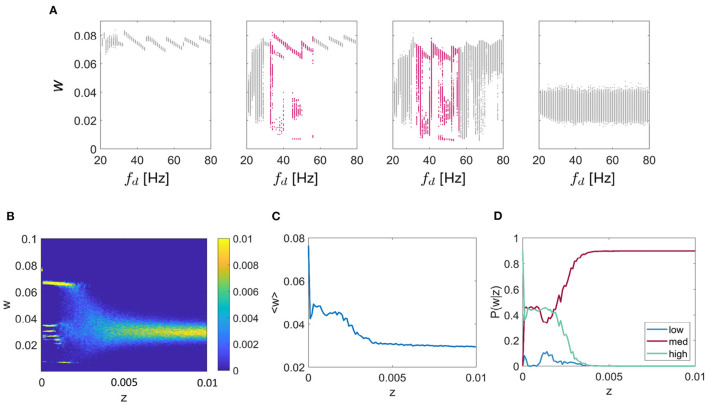
Multi-unit simulation. Results for the 10-unit network are shown. **(A)** The effect of driving frequency and noise on the synaptic strength *w* for a network tuned to 12 Hz resonance frequency (*f*_*r*_) with a range of inputs (*f*_*d*_) between 20 and 80 Hz for the noise values z = 0 (left), *z* = 0.0001, *z* = 0.001, *z* = 0.1 (right). For additive noise values shown here of *z* = 0.01, and *z* = 0.001, there are regions of the plot that show meta/ multistability (highlighted in pink). **(B)** The effect of noise on multistable plasticity with *f*_*r*_ = 12 Hz and *f*_*d*_ = 48 Hz, chosen so as to highlight the multistable behavior for a range of additive noise (*z*). **(C)** The mean connection strength over all trials as a function of the noise with *f*_*r*_ = 12 Hz and *f*_*d*_ = 48 Hz, fixed as above. **(D)** *P*(*w*|*z*) is given for each of the 3 connection strength states “low,” “mid,” and “high,” again for the case *f*_*r*_ = 12 Hz and *f*_*d*_ = 48 Hz as above.

Thus far, we have characterized synaptic multistability in a two-unit WC system. To explore how the results apply to larger systems we extended the model to include ten fully-connected units. The network was tuned to exhibit resonance at 12 Hz. Initially, we tested the effect of driving frequency on the final connection strength between each pair of units, Figure 6A for noise values of *z* = 0 (left), *z* = 0.0001, *z* = 0.001, and *z* = 0.1 (right) for values of *f*_*d*_ between 20 and 80 Hz, chosen to highlight the region of multistable behavior. It can be seen that as was observed in the two-unit system, in the two central plots there are regions of multistability (highlighted in pink), although the behavior of the network is noisier. Next, we fixed *f*_*d*_ = 48*Hz*, as in the 2-unit example described earlier and systematically tested the effect of additive noise on the connection strength shown in Figure 6B. We observed a similar pitchfork pattern as in the 2-unit network, with the main difference that for the 10-unit network the mid level is split into multiple sub-levels of the connection strength. In Figure 6C, we show the ensemble average connection strength as a function of additive noise and recognize the same pattern in the large network as we saw previously; lower levels of noise facilitate higher connection strengths. Finally, we give *P*(*w*|*z*) for each of the three states “low,” “mid,” or “high”—noting that all branches of the “mid” level are included together—and again conclude that for lower levels of noise; the probability of reaching the highest weight is maximized (see Figure 6D).

**Figure 7:**
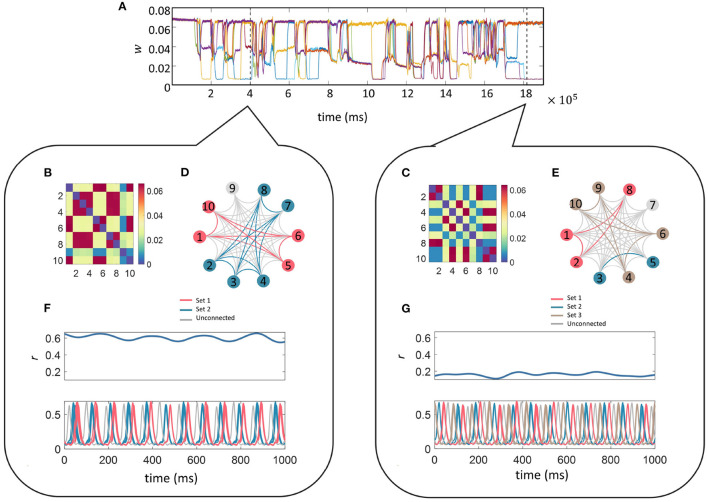
Multi-unit simulation. The parameters for the 10-unit network were fixed as *f*_*r*_ = 12 Hz, *f*_*d*_ = 48 Hz, and *z* = 0.015. The top trace **(A)** illustrates the temporal evolution for a selection of *w*; here we show the connections between unit 1 and the other 9 units (units are connected to everyone apart from themselves) as a function of time. The connectivity matrices containing the final values for w between the 10 units related to the 1,000 ms of the simulation starting at each of the dashed lines in **(A)** is given in the matrices **(B, C)** within each bubble (left and right). Sets of units connected *via* “high” *w* during the selected time frame are color-coded and indicated on the star diagrams **(D, E)** for a visual representation of the transient highly-connected sub-networks. Finally, the global coherence (*r*(*t*), top panel) and the time series (lower panel) for each of the 10 units within the selected time-frame color coded to match the star diagram to illustrate the high coherence of activity within each sub-network are shown in **(F, G)**. The sub-networks switch and reorganize many times during the simulation and it can be seen that they have been rearranged between these two-time points.

Finally, we fixed the ten-unit network with parameters *f*_*r*_ = 12 Hz, *f*_*d*_ = 48 Hz, *z* = 0.015 and initially plotted the time evolution of *w* between unit 1 and the other 9 units (units are connected to everyone apart from themselves) as a function of time in Figure 7A. It can be seen that the connectivity strengths organize themselves into the same three stable states as was observed in the smaller network. Switching between the states occurs spontaneously for all connections at the same time which is followed by a reorganization of the connection strengths. To illustrate this point we analyzed the connectivity matrices and time series for two distinct time points of the simulation indicated by the two dashed lines in Figure 7A (see bubbles).

Each self-contained bubble (left and right) relates to a 1,000 ms time period indicated by the dashed lines. The connectivity matrices contain the final value for *w* between all 10 units within the selected time frame. Sets of units connected *via* “high” *w* are color-coded as indicated on the star diagrams for a visual representation of the transient nature of the highly-connected sub-networks mimicking transient functional networks observed in the brain during task performance or at rest.

The time-series for each of the 10 units within the selected time-frame are plotted color-coded to match the star diagram to illustrate the high coherence between units within each sub-network. Traces from the same sets appear so highly correlated that they completely overlap. The sub-networks switch and reorganize many times during the simulation and it can be seen that the connectivity architecture has been completely rearranged between these two time points. For instance, during the first period (left bubble), Units 1, 5, 6, and 10 (pink) are connected *via* “high” strength connections. Their temporal traces are completely overlapping in the lower trace. In the second period (right bubble), Unit 1 is now connected to Units 2, and 8 *via* a “high” strength connection and the temporal correlation between Unit 1 and its previous sub-network has been lost. The difference in the overall level of connectivity in the network between the state at the left and right bubbles is also reflected the global coherence as quantified by *r*(*t*) in the lower panels.

## 4. Discussion

Neural ensembles residing within disparate brain regions oscillate across a wide range of frequencies. To orchestrate complex brain functions, these sub-networks may transiently connect with each other across multiple spatial and temporal scales dependent on the task at hand (Fries, 2005). Multistability has been proposed as a potential mechanism underlying this process (Kelso, 2012; Tognoli and Kelso, 2014; Alderson et al., 2020), and is thought to play a crucial role in the brain in the context of numerous cognitive processes, including perception, binocular rivalry, and auditory stream segregation (Atteneave, 1971; Leopold and Logothetis, 1999; Winkler et al., 2012; Feudel et al., 2018; Hramov et al., 2018). The existence of multistable regimes has also been observed in resting-state data which switches between different transient network states. In this paper, we tested how different multistable regimes affect frequency-dependent plasticity, and what the role of noise is in this process.

Initially, we tested the effects of driving frequency and additive noise on the connection strength in a two-unit model. We found that for low levels of additive noise (*z* < 0.01), and for driving frequencies within a specific range, a region of multistability existed where the final connection strength could take one of three values (referred to as “low,” “mid,” or “high”). We tested this effect on variations of the tuning of the model to exhibit resonance at 4, 8, 12, and 23 Hz, loosely corresponding to the delta, theta, alpha, and beta oscillatory regimes in the brain. The regions of multistability span a range of driving frequencies between approximately twice the resonance frequency of the system and ten times this amount (Figure 2). When the level of noise passed a certain threshold, the multistability was abolished. The greatest connection strengths were found to be at driving frequencies close to the resonance frequency of the system [confirming the results previously found in Lea-Carnall et al. (2017) in the case when both units received periodic stimulation as was the case here] which is increasingly apparent at higher levels of additive noise.

We next explored in more detail the effect of adding noise to models with a specific combination of resonance and driving frequencies chosen to highlight the multistable regions (Figure 3). The resonance frequencies were fixed as before and the driving frequency was chosen to be a multiple of this by either 4 or 5 times to focus on a multistable region of the space. We varied the additive noise between *z* = 0 and *z* = 0.02 and found that for low noise, *z* < 0.003, there was a clear multistable region for which the connection strength took one of the three values described previously. As the additive noise approached a critical value (approximately *z* = 0.005), the regions of multistability become less well-defined until finally, the multistable states collapse, leading to a final value of the ECS within a range of values centered around the “mid” strength for the weight.

There has been significant computational work recently aiming to elucidate how multistable brain-states aid cognition. For example, Golos *et al*. used a whole-brain model to determine that the presence of multistable attractors in the model determined its ability to generate transient patterns of activity which were related to resting state MRI dynamics (Golos et al., 2015). Computational modeling studies have also found that neural systems operating close to criticality maximize their processing potential (see Cocchi et al., 2017 for a review). Orio *et al*. showed that a conductance-based model with chaotic dynamics exhibited multistable dynamics within a specific range of connectivity strengths (Orio et al., 2018). Furthermore, the authors showed, in agreement with our results, that in networks of non-chaotic nodes, low levels of noise-induced multistable behavior while higher noise levels abolished the multistable regime. Litwin-Kumar and Doiron used a network model which included synaptic plasticity and homeostasis, and were able to replicate the stable and transient formation of neural assemblies that reflected previously experienced stimuli (Litwin-Kumar and Doiron, 2014). Finally, Pisarchik *et al*. found the co-existence of three different oscillatory states in a network of 2 interconnected units which were related to the value of the (fixed) coupling strengths (Pisarchik et al., 2018). In a recent study focused on multistability in a neural network with STPD, Madadi Asl et al. (2018a) found that the initial distribution of the synaptic weights gave rise to different connectivity patterns. We did not find any difference in the outcome when the initial values for the weights were varied (results not shown). However, one important difference from our study is that Madadi Asl et al. included axonal propagation delays which were omitted from the current model. Furthermore, when the length of axonal pathways cannot be neglected, propagation delays can have an important role on neural coherence, as has been explored int the context of whole-brain dynamics (Ton et al., 2014; Petkoski and Jirsa, 2019). Additionally, synaptic delays together with synaptic scaling have been shown to modulate synchrony in the aging brain, where alpha-band phase locking is maintained over the lifespan presenting an age-related shift in peak alpha frequency (Pathak et al., 2022). This is an area for further study in which synaptic dynamics are incorporated into larger networks.

A major aim of this work was to provide predictions that could be tested in plasticity experiments. In particular, a direct interpretation of our results indicate that it may be possible to optimize synaptic strength from the interplay between noise and driving frequency. With this in mind, we calculated the mean connection strength between the units as a function of noise for the same four combinations of resonance and driving frequency described previously and we found that for low values of noise (*z* < 0.005), the mean connection strength across all trials was maximized. We also found that the probability of the connection strength obtaining the “high” value was maximized for a non-zero, low-level intensity of the additive noise (see Figure 4).

To characterize the dynamics of the model for the different stable states of the connection strength, we compared the scaled truncated correlation γ*C*_Δ_ (see Methods), to the coarse-grained connection strength, $\bar{w}$. We found that the connection strength regularly switched between the three states (“low,” “mid,” and “high”) and that the temporal evolution of $\bar{w}$ closely matched the truncated correlation, indicating that the latter provided an excellent estimation of connection strength. We also reported the *PLV*, a measure of coherence between the phases of two signals and note that this is generally close to 1, indicating phase locking of the signals, apart from at the points where “switching” occurs where the *PLV* is momentarily reduced. Therefore, the three stable states, “high,” “mid,” and “low” corresponded to a decreasing level of phase coherence between the two units. This is further evidenced in the phase-space trajectories of the excitatory activity of one unit against the other within each of the three regimes. We found that when the weight is in the “low” state, the activity of the two units is anti-correlated (see Figure 5C). Whereas, when the connection strength is “high” the behavior of *E*_1_ vs. *E*_2_ shows a high degree of correlation (see Figure 5D).

Finally, it is important to understand whether the behavior described so far for a small two-unit model extends to larger networks. For this, we implemented an all-all connected ten-unit network with fixed resonance of 12 Hz. We first repeated the investigation into the effect of driving frequency on connection strengths between the units and found that multistable regions of the connection strength did exist for specific ranges of driving frequency and additive noise, as was the case for the two-unit model (see Figure 7). Next, we fixed the driving frequency to 48 Hz (as in the smaller network) and tested the effects of increasing additive noise on the model output. We found that the same multistability emerged as for the smaller network, but in this case, instead of the original 3 branches to the pitchfork for *z* < 0.005, the “mid” branch splits into further sub-levels. Interestingly, in the larger network, we also found that the lowest intensities of the additive noise resulted in the largest mean connection strength. Additionally, the probability of obtaining the “high” connection strength was maximized for low levels of noise, as in the smaller two-unit network. We also observed the same switching between states in the large network as the simulation evolved through time. The connection strengths reorganized themselves multiple times allowing smaller highly connected subnetworks to form transiently, providing a mechanism of functional connectivity in the model.

The findings presented here suggest that plasticity outcomes can be enhanced by specific levels of additive noise as a consequence of an interaction between noise and multistability. This prediction could be tested in multiple ways experimentally; the effect of extrinsic noise on plasticity and learning could be tested by controlling the intensity of noise added to a stimulus designed to elicit plasticity in a targeted brain region. As an example of this, Xie et al. (2018) added moderate levels of noise to a visual stimulus presented at a range of frequencies and found that synaptic potentiation was increased for some stimulation frequencies with the addition of noise compared to the same stimulus without noise. In the somatosensory system, a similar protocol to that followed by Lea-Carnall et al. (2017) could be implemented in which mechanical noise is added to the stimulus. Probing different sensory areas would provide a framework to understand how plasticity can be modulated by the interplay between stimulus and resonant frequencies under different levels of noise intensity. We suggest that, given the growing body of evidence indicating that noise plays a non-trivial role in biasing multi-state synapses, noise should be accounted for in future plasticity studies.

## Data availability statement

The raw data supporting the conclusions of this article will be made available by the authors, without undue reservation.

## Author contributions

CL-C and MM: conceptualization, data curation, formal analysis, investigation, methodology, visualization, writing—original draft, and writing—review and editing. LT: investigation, methodology, writing—original draft, and writing—review and editing. All authors contributed to the article and approved the submitted version.
