# Supplementary material for: Noise-modulated multistable synapses in a Wilson-Cowan-based model of plasticity
Source: Front Comput Neurosci. 2023 Feb 2;17:1017075. doi: 10.3389/fncom.2023.1017075 (PMC9931909; doi:10.3389/fncom.2023.1017075)
Supplement: Supplementary file 1 [file Data_Sheet_1.PDF]

Supplementary Material

1 SUPPLEMENTARY TABLES AND FIGURES

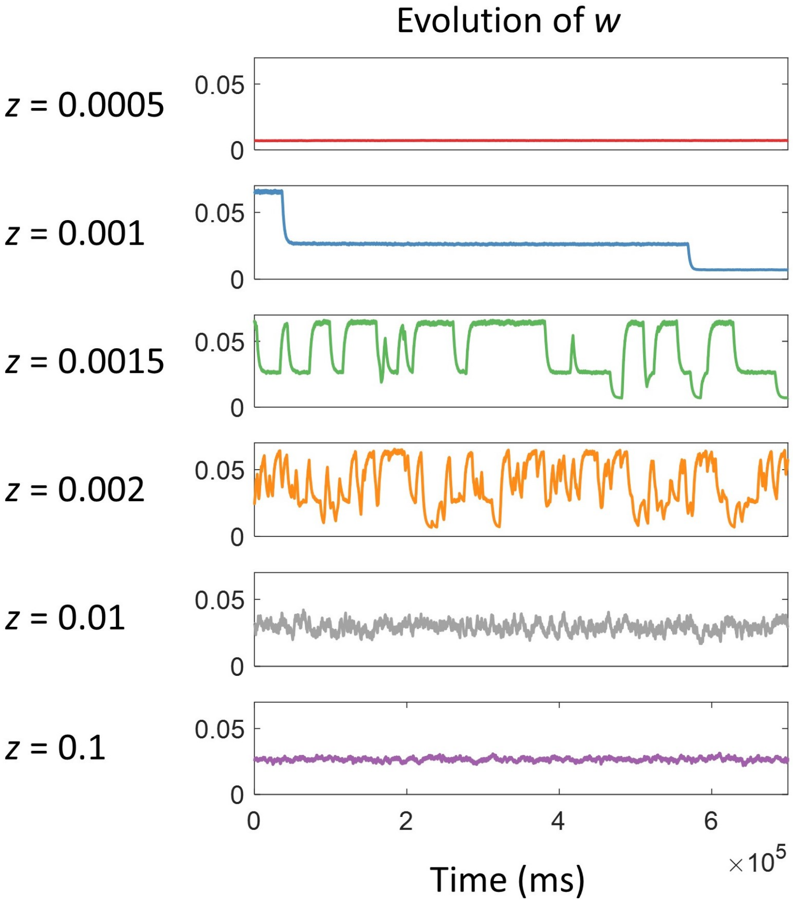

Figure S1. Effect of noise on the temporal evolution of w. Here we examine a two-unit model with the conditions f_r = 12 Hz, f_d= 48 Hz and a range of additive noise values, z. We observe that for low noise (z=0.0005) the weight reaches a steady state and remains there; for intermediate z (z between 0.001 and 0.002) the weight switches between the states more rapidly with increasing z; for z greater than the critical point z_c where the multistable states collapse (compare to Fig. 3).
